# Impact of Various Beam Parameters on Lateral Scattering in Proton and Carbon-ion Therapy

**Published:** 2015-12-01

**Authors:** M. Ebrahimi Loushab, A.A. Mowlavi, M.H. Hadizadeh, R. Izadi, S.B. Jia

**Affiliations:** 1 Physics Department, Faculty of Sciences, Ferdowsi University of Mashhad, Mashhad, Iran; 2 Physics Department, School of Sciences, Hakim Sabzevari University, Sabzevar, Iran; 3 ICTP, Associate Federation, Medical Physics Section, Trieste, Italy; 4 Physics Department, University of Bojnord, Bojnord, Iran

**Keywords:** Geant4, Proton Therapy, Carbon-ion Therapy, Bragg Peak, Multiple Scattering, Hadronic Interaction, Lateral Dose

## Abstract

**Background:**

In radiation therapy with ion beams, lateral distributions of absorbed dose in the tissue are important. Heavy ion therapy, such as carbon-ion therapy, is a novel technique of high-precision external radiotherapy which has advantages over proton therapy in terms of *dose locality* and *biological effectiveness*.

**Methods:**

In this study, we used Monte Carlo method-based Geant4 toolkit to simulate and calculate the effects of energy, shape and type of ion beams incident upon water on multiple scattering processes. Nuclear reactions have been taken into account in our calculation. A verification of this approach by comparing experimental data and Monte Carlo methods will be presented in an upcoming paper.

**Results:**

Increasing particle energies, the width of the Bragg curve becomes larger but with increasing mass of particles, the width of the Bragg curve decreases. This is one of the advantages of carbon-ion therapy to treat with proton. The transverse scattering of dose distribution is increased with energy at the end of heavy ion beam range. It can also be seen that the amount of the dose scattering for carbon-ion beam is less than that of proton beam, up to about 160mm depth in water.

**Conclusion:**

The distortion of Bragg peak profiles, due to lateral scattering of carbon-ion, is less than proton. Although carbon-ions are primarily scattered less than protons, the corresponding dose distributions, especially the lateral dose, are not much less.

## Introduction

Energy deposition of charged particles like protons or heavier ions increases with penetration depth and reaches a maximum just before the end of range, the so-called *Bragg peak*. Treatment of cancer with ionizing ions is known as “hadron therapy”, which uses hadrons, such as neutrons, protons, light ions and heavy ions. Heavy ion therapy, such as carbon-ion therapy, is a novel technique of high-precision external radiotherapy which has advantages over proton therapy in terms of *dose locality* and *biological effectiveness* which is addressed in many references[1, 2]. In this study, the Monte Carlo code Geant4 has been used. Geant4 is an object-oriented based simulation toolkit used for transport particles which is able to simulate the interaction of particles with matter and the production of secondary particles[3-5].

We will focus on electromagnetic and Hadronic interactions in the present work. There are currently 28 “packages” as Physics lists, available in Geant4.9.6, but the user must have the know-how to select the appropriate model. Given the toolkit nature of Geant4, a choice of physics process is available. The choices offer either different details of physics modelling or different physics modelling descriptions. It is the choice of the user to decide how much detail in the physics modelling is needed, weighing the detail against CPU performance. A comparison between different nuclear models has been made and reported by Lechner et al.[6], Kameoka et al.[7] and many references[8, 9]. However, one study indicates that secondary particle production, especially the positron emitters, is very sensitive to change nuclear models[10].

## Material and Methods

**Figure 1:**
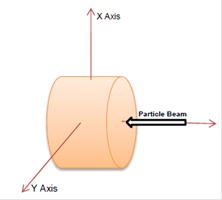
Geometry of the phantom, the cylindrical water phantom extending along the z-axis

To assess the influence of configuration of the incident ion beam on transverse distribution of the absorbed dose, Monte Carlo-based simulations were conducted by using toolkit Geant version 4.10.1. Geometries dictated to the toolkit were a cylindrical water phantom of 20cm (radius) × 30cm (height), as the target sitting on the xy-plane with the z-axis as its axis of symmetry, shown in Figure 1. The primary particle source, emitting protons or carbon-ions, were in the proximity of the phantom base on the z-axis. Mono energetic pencil beams of protons (50, 100, 150 and 200 MeV), and carbon ions (1101, 2240, 3418, and 4632 MeV) “presumably” hit the phantom. These energy choices should, theoretically, establish a one-to-one correspondence between the ranges of the two projectiles in mind. For example, the range of 50 MeV protons in water is, approximately, equal to the range of 1101 MeV carbon ions in this material. What we have done is theoretical, but not a “long shot” in practice! GSI in Germany is capable of producing ions of 48-430 MeV/u.

The number of events in this simulation is typically of the order 107 for protons and 106 for carbon no I, respectively. As for the reason behind selecting 10 times less carbon ions than protons for primary events, one should bear in mind that more secondary particles are produced by carbon ions than those by protons[19]. The numbers we have chosen, give almost the same standard errors for protons and carbons. Besides, carbon ions consume more calculation time than protons; selection of less number of carbon ions makes our calculations less lengthy, without sacrificing the accuracy of the results.

To estimate the energy absorbed in the phantom, we subdivided the main cylinder into small parallel cylindrical cells of radius *r* and height *h*, in which *h* remains constant at 0.1mm, and *r* varies from 0.1 to 1.0 mm in 0.1mm steps.

We will be using *G4Em Standard Physics- option3* for electromagnetic interactions and *Quark-Gluon String Pre-compound* (QGSP) model including Elastic for elastic collision and Binary for inelastic collision[11], and *local-ion-ion-inelastic* for ion inelastic interactions for Hadronic interactions which are recommended in Hadron therapy project[12] and[13]. The contribution of secondary neutrons in dose calculation is important. About two percent of the total[14], in physics list, package of G4Neutron HP Builder is included. We set the production threshold of secondary particles (according to Geant4 terminology “SetCut”) to 100µm for electron, positron, gamma and proton, which is less than the minimum cell widths. It should be noted that if the SetCut is reduced, then the number of secondary particles, in particular electrons, increases and greatly affects calculation time[15].

We used a 50 MeV proton beam and calculated the total energy (due to primary and secondary particles, especially, delta electrons that are not tracked and are cutoff) deposited in each of the small cells mentioned above. The cell radii were then changed, step by step, from 0.1mm to 1mm and the corresponding Bragg peaks were found. Similar calculations were carried out for 100, 150 and 200 MeV energies. 

## Results and Discussion

**Figure 2:**
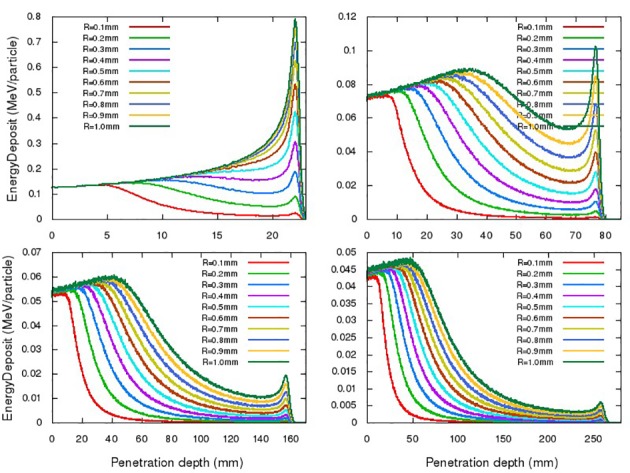
Beam energy effects on Bragg peak profile for protons; (Top-Left) E_p_= 50 MeV, (Top-Right) E_p_= 100 MeV, (Bottom-Left) E_p_= 150 MeV, and (Bottom-Right) E_p_= 200 MeV.

The influences of energy beam on lateral scattering and on the shape of simulated Bragg peak and the dose distribution in cells were investigated. The resulting Bragg peak profiles are shown in Figure 2. Simulations for proton beams show that for energies higher than 50 MeV, the Bragg peaks for cells smaller than 1-mm dimensions “fade away”. With increasing beams energy, “fading away” increases.

**Figure 3:**
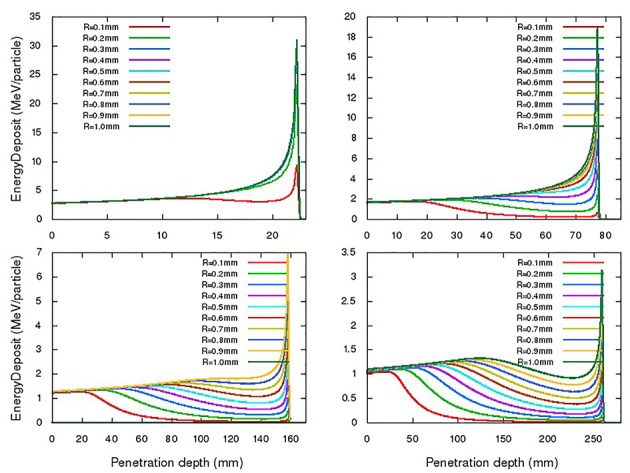
Beam energy effects on Bragg peak profile for carbon-ions: (a) E_C_= 1101 MeV, (b) E_C_= 2240 MeV, (c) E_C_= 3420 MeV, and (d) E_C_= 4630 MeV.

The intensities also drop with energy increase strongly (see Figure 2). The beam energy effects on Bragg peak profile for carbon-ions at Ec=1101, 2240, 3420 and 4630 MeV are presented in Figure 3. In calculating the absorbed energy of proton beams, relative standard errors in the cells near the front face of the phantom and those near Bragg peaks change from 0.1 to 0.4 percent. For carbon-ion beams, they go from 0.1 up to 0.3 percent. Smallness of the calculated standard deviations prevents the appearance of the error bars in the figures.

As compared to protons, in the case of carbon-ions, the Bragg peak shape is preserved up to ~3400 MeV (280 MeV/nucleon). Moreover, the distortion of Bragg peak profiles is less than proton due to the lateral scattering of carbon-ion.

**Figure 4:**
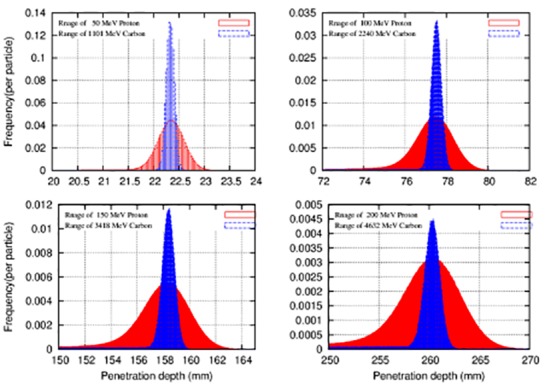
Differential distributions for carbon-ion and proton ranges

As it is well known, lateral scattering can increase range uncertainties of proton and carbon-ion beams. Mean range is defined as the absorber thickness that reduces the light ion particles count rate to exactly one-half of its value in the absence of the absorber[16]. The differential range-distributions of primary particles, protons and carbon-ions are shown as probability vs. penetration depth plots in Figure 4. Since, at identical energies, carbon-ion range in matter is much less than that of proton, we increase carbon energy until its range reaches that of proton. For example, the 1101 MeV and 4632 MeV carbon-ranges are close to those of the 50 MeV and 200 MeV protons, respectively.

It can be seen that they, very much, show a “normal” or Gaussian behavior. Therefore, we have fitted the simulated results with a Gaussian function as follows:

$$f(x)=\frac{1}{\sigma \sqrt{2\pi }}e^{\frac{{\left(r-\mu \right)}^{2}}{{2\sigma }^{2}}}\to Range=\mu \pm \sigma \;(1)$$

Where *μ* is the mean range and *σ* is a parameter related to the full width at half maximum as FWHM= 2*σ* √2*ln* (2). Values obtained for these parameters in different energies of proton and carbon-ion are listed in Table 1. We have also quoted the mean proton range, reported by ICRU, in the last column of this table for comparison[17].

**Table 1:** Values of fitted parameters in a Gaussian function for different energy of proton and carbon.

**Beam Particles**	**Energy** **(MeV)**	**This work** **Mean Range (mm)**	**σ(mm)**	**Mean Range (mm)**
**Proton**	50	22.35	0.25	22.08
	100	77.44	0.79	76.59
	150	158.22	1.79	156.8
	200	260.28	2.81	258.1
**Carbon-ion**	1101	22.33	0.08	22.35
	2240	77.51	0.24	77.98
	3418	158.39	0.46	158.69
	4632	260.32	0.72	260.72

This table shows whereas, with increasing particle energies, the width of the Bragg curve becomes larger, the width of the Bragg curve decreases with increasing mass of particles. This is one of the advantages of carbon-ion therapy to treatment with proton.

**Figure 5:**
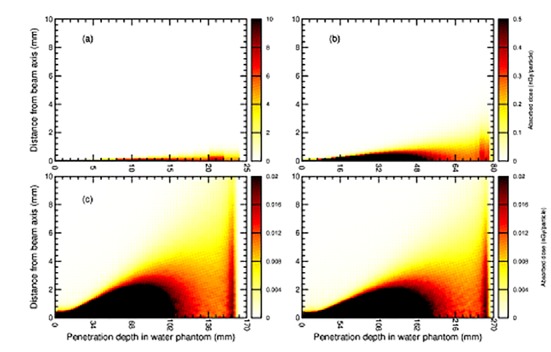
Proton 2D dose profiles: (Top-Left) E_p_= 50 MeV, (Top-Right) E_p_= 100 MeV, (Bottom-Left) E_p_=150 MeV, and (Bottom-Right) E_p_= 200 MeV (Dose scales may differ in each case).

**Figure 6:**
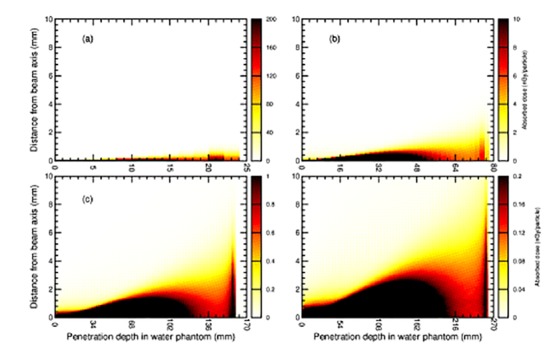
The 2D dose profile for carbon beam: (Top-Left) E_C_= 1101 MeV, (Top-Right) E_C_= 2240 MeV, (Bottom-Left) E_C_= 3420 MeV, and (Bottom-Right) E_C_= 4630 MeV (Dose scales may differ in each case).

In hadron therapy, the accurate determination of the 2D absorbed dose distribution in lateral scattering is very important. Like all other calculations done in this work, “USERHOOK” method is used to retrieve the radial dose distribution as a function of penetration depth. Appropriate energies, as mentioned before, were chosen for relevant ions[18]. The results obtained for the 2D dose profiles for proton and carbon-ion beams with different energies are shown in figures 5 and 6. As can be seen, the transverse scattering of dose distribution is increased with energy at the end of heavy ion beam range. It can also be seen that the amount of the dose scattering for carbon-ion beam is less than that of proton beam, up to about 160mm depth in water.

Since transverse distribution of the dose in hadron therapy is important, in treatment planning, the lateral beams behavior for proton beams and carbon ions should be considered. However, lateral scattering in carbon ion beams is less than that from proton beams, but in higher energy beams, by taking into account secondary particles, the lateral distribution dose in deep tissues is increased. For this reason, in the depth of more than 160mm, the lateral scattering is greater than proton beams due to the effect of secondary particles for carbon ions[19, 20].

## Conclusion

Although carbon-ions are primarily scattered less than protons (Figures 4), the corresponding dose distributions especially the lateral dose, are not much less (see figures 5 and 6). This is due to the secondary particles generated by carbon-ions; an absent effect or at least less abundant than in the proton case. Our calculations show that this is valid up to about 160mm depth in water. For deeper penetrations or seated tumors, protons seem to be more effective.
